# Outcomes of a Standardized Protocol on the Management of Acute Type A Aortic Dissection: A Retrospective Cohort Study

**DOI:** 10.1055/s-0045-1809170

**Published:** 2025-05-13

**Authors:** Supitchaya Philippoz, Dionysios Adamopoulos, Tornike Sologashvili, Mathieu van Steenberghe, Jalal Jolou, Christoph Huber, Mustafa Cikirikcioglu

**Affiliations:** 1 Division of Cardiovascular Surgery, Department of Surgery, Geneva University Hospitals and Faculty of Medicine, Geneva, Switzerland; 2 Division of Cardiology, Department of Medical Specialties, Geneva University Hospitals and Faculty of Medicine, Geneva, Switzerland

**Keywords:** acute Type A aortic dissection, aortic surgery, acute aortic syndrome

## Abstract

**Background:**

Acute Type A aortic dissection (AAAD) is a life-threatening condition, with surgery being the recommended treatment. However, there is ongoing debate regarding the optimal surgical procedure. This study aimed to evaluate the impact of implementing a standardized protocol, introduced in our institution in 2016, on AAAD management.

**Methods:**

A retrospective cohort study was conducted involving patients treated surgically for AAAD between 2010 and 2021 in our department. Patients were divided into two groups: those who underwent surgery before 2016 using operator-dependent techniques, and those who underwent surgery starting in 2016 using a standardized protocol.

**Results:**

A total of 104 patients were included in this study. The mean age was 66.5 ± 11.4 years and 55.8% were male. Demographics and preoperative data were similar in both groups. Arterial and venous cannulation site of both groups were different ( *p* < 0.001): femoral artery and vein cannulation for group 1 versus subclavian artery and central venous canulation for group 2. Alone ascending aorta replacement versus ascending aorta plus hemiarch replacement were the preferred techniques in groups 1 and 2, respectively ( *p* < 0.001). Hypothermic circulatory arrest and cerebral perfusion were largely performed in group 2 compared with group 1 ( *p* < 0.001). The total time of surgery, the cardiopulmonary bypass, and aortic cross-clamping times were longer in group 2 ( *p* < 0.05). Both groups had similar rates of postoperative complications, except for late reoperation and aortic dilatation rates, which were less frequent in group 2 ( *p* < 0.05).

**Conclusion:**

The implementation of a standardized institutional protocol can transform AAAD surgery from a “surgeon-tailored” to a “ patient-tailored” approach. The use of a standardized protocol in our institution resulted in a significant reduction of aortic reoperation and aortic dilation rates, suggesting that the introduction of standardized protocols in low-volume centers may improve AAAD management.

## Introduction

Aortic dissection (AD) is a type of acute aortic syndrome (AAS), a spectrum of severe and life-threatening aortic pathologies. AD occurs when an intimal tear of the aortic wall causes accumulation of blood between the intima and media of the aorta, forming a false lumen parallel to the native aortic lumen. After the onset of symptoms, diagnosis of AD is categorized as acute within 14 days, subacute between 14 and 90 days, and chronic (although less common) after 90 days. 1 The annual incidence of AD is between 4 and 6 cases per 100,000 individuals. 2

The Stanford Classification System, the most commonly adopted system for AD classification, distinguishes between two main types: Stanford Type A, which involves the ascending aorta, or Stanford Type B, which only affects the descending aorta. While Stanford Type B usually only require early medical treatment, Stanford Type A dissections generally require surgical intervention. 1 2 Acute Type A aortic dissection (AAAD) is the most lethal form of dissection. Without prompt treatment, patients with AAAD can die from serious complications like pericardial tamponade, organ malperfusion, or myocardial ischemia lumen. 1 2 Without surgical intervention, mortality rates for AAAD reach 50% within the first 48 hours. 1

Surgery is the recommended treatment for AAAD, yet a consensus on the optimal protocol for management of AAAD has not been reached. Standardized protocols for AD surgery could help ensure speed and efficiency, key parameters on which surgeons can act on to improve patient survival rates. In 2016, our institution introduced a standardized protocol, replacing the previously used operator-dependent approach. The study aims to investigate the impact of implementing a standardized surgical protocol on AAAD management, with a focus on postoperative complications, causes and rate of reoperation, and survival.

## Materials and Methods

### Data Collection

We conducted a retrospective review of our institution's electronic medical records to identify all patients who underwent surgery for AAAD between 2010 and 2021 at our institution. Data on preoperative, perioperative, and postoperative parameters were collected. These parameters included demographic information (gender, age), patient background (medical and surgical history, risk factors), clinical presentation, diagnosis methods, details on surgical procedure, complications, and survival outcomes.

### Patients

A total of 3,155 surgeries were registered between 2010 and 2021, of which 122 operations were for an AD. Of these, 118 were for Type A and 4 for Type B dissections. After excluding cases of subacute or chronic AAAD, a total of 104 procedures for AAAD were included for analysis in this study. Patients were divided into two groups for comparison: 58 patients who underwent surgery before 2016 using operator-dependent techniques and 46 patients who underwent surgery starting in 2016 using a standardized protocol.

### Surgical Technique

Surgery aims to replace the affected part of the aorta with a synthetic graft. At the same time, an insufficient aortic valve can be restored or replaced, and coronary arteries may be reimplanted on the aortic graft or preserved. If the supra-aortic vessels are affected, they can be replaced as well. Ultimately, the extent of the surgical procedure depends on the extent of the dissection. Surgery will be more complicated and take longer when more surrounding structures are involved. The intervention is even more challenging as we consider the adjunctive measures: cannulation, hypothermic circulatory arrest (HCA), and perfusion of vital organs.

When the dissection is limited to the ascending aorta, ascending aorta replacement alone can be performed, which is the easiest and quickest technique to perform. If the AD involves proximal structures, valve-sparing techniques such as the Yacoub and Tirone-David procedures are used to either replace or repair the aortic root and the aortic valve. The Yacoub procedure consists of an aortic valve remodeling, so as to replace the aortic sinus with a tailored graft, 1 whereas the latter involves reimplantation of the aortic valve within a graft, which is sutured on the aortic annulus. 1 If needed, a Bentall procedure using a composite graft 3 can unite the aortic valve, the aortic root, and the ascending aorta. A hemiarch or total-arch replacement is performed when the AD extends to the aortic arch and beyond. A frozen elephant trunk technique can achieve more extensive distal repair through graft replacement of the ascending aorta and aortic arch associated with a stent-graft placed in the descending aorta. 1

### Statistical Analysis

Frequency or mean were calculated for each parameter. Categorical variables are expressed as frequency (percentage), whereas continuous variables as mean ± standard deviation. Continuous variables were compared using Student's *t* -test. Categorical variables were compared using χ ^2^ test (Fisher's test) or Fisher's exact test when sample size < 5. Survival analyses were performed with the Kaplan–Meier estimator. A *p* -value ≤ 0.05 was considered statistically significant. All data were analyzed using SPSS software.

### Ethical Consideration

Prior to accessing their medical records, selected patients were provided with an informative letter explaining the study and requesting their participation. The letter explicitly stated that individuals who did not wish to be included could respond via email or traditional mail, and their silence would be interpreted as consent. In our study, not patients opted out. The identity of included patients was anonymized using a random code during data collection. This study adheres to the regulatory requirements of the HRA (Human Research Act) and HRO (Ordinance on Human) and has received approval from the local ethics committee.

## Results

### Patient Demographics

Of 104 patients included, 55.8% were male and mean age on the day of surgery was 66.5 ± 11.4 years. Patient and preoperative characteristics are summarized in Table 1 . Women were statistically significantly older than men, with a mean age of 71.6 ± 7.2 versus 62.4 ± 12.4, respectively. Over half of patients had hypertension (55.8%). A total of 4 patients had known vascular inflammation disease, 3 of which had Horton disease and 1 had Wegener's disease. A total of 9 patients had previous heart surgery and 3 patients had iatrogenic AD secondary to cardiac catheterization.

**Table 1:** Demographics

Parameter	Overall ( *N* = 104)	Group 1 ( *N* = 58)	Group 2 ( *N* = 46)	*p* -Value
Gender				
Men	55.8% (58)	58.6% (34)	52.2% (24)	–
Women	44.2% (46)	41.4% (24)	47.8% (22)	–
Mean age (y) ± SD	66.5 ± 11.4	65.2 ± 11.5	68.0 ± 11.1	0.244
Men	62.4 ± 12.4	–	–	–
Women	71.6 ± 7.2	–	–	–

Abbreviation: SD, standard deviation.

### Clinical Presentation

Table 2 lists clinical signs and symptoms. Most patients (75.0%) had an abrupt onset of symptoms, with pain being the most common complaint (87.5%). Chest pain was most common, followed by abdominal and back pain ( Table 3 ), and the average intensity of pain was reported by patients to be 7.9 ± 2.1 out of 10. A total of 14 patients (13.9%) had a shock or cardiorespiratory arrest, and 34 patients (32.7%) presented neurological symptoms, mostly with sensory–motor deficits.

**Table 2:** Clinical presentation

Parameter	Overall ( *N* = 104)	Group 1 ( *N* = 58)	Group 2 ( *N* = 46)	*p* -Value
Abrupt onset of symptoms	75.0% (78)	70.7% (41)	80.4% (37)	0.254
Pain	87.5% (91)	84.5% (49)	91.3% (42)	0.296
Syncope	26.9% (28)	24.1% (14)	30.4% (14)	0.472
Nausea/vomiting	19.2% (20)	19.0% (11)	19.6% (9)	0.939
Dyspnea	17.3% (18)	15.5% (9)	19.6% (9)	0.588
Cough	2.9% (3)	3.4% (2)	2.2% (1)	1.000
Neurologic symptoms	32.7% (34)	31.0% (8)	34.8% (16)	0.686
Shock/cardiorespiratory arrest	13.9% (14)	12.7% (7)	15.3% (7)	0.718
Physical examination				
Hypotension	28.8% (30)	25.9% (15)	32.6% (15)	0.451
Diaphoresis	21.2% (22)	19.0% (11)	23.9% (11)	0.539
Pallor	13.5% (14)	17.2% (10)	8.7% (4)	0.205
Tension asymmetry	11.5% (12)	10.3% (6)	13.0% (6)	0.669
Bradycardia	12.5% (13)	10.3% (6)	15.2% (7)	0.456
Pulse deficit	13.5% (14)	13.8% (8)	13.0% (6)	0.911
Auscultated aortic murmur	8.0% (8)	5.4% (3)	11.4% (5)	0.295
Time between first symptoms and arrival in the ED, mean ± SD (h)	22.5 ± 63.8	19.9 ± 61.6	24.8 ± 66.4	0.748

Abbreviations: ED, emergency department; SD, standard deviation.

**Table 3:** Pain

Parameter	Overall ( *N* = 91)	Group 1 ( *N* = 49)	Group 2 ( *N* = 42)	*p* -Value
Location				
Chest pain	72.5% (66)	73.5% (36)	71.4% (30)	0.828
Back pain	8.8% (8)	4.1% (2)	14.3% (6)	0.137
Abdominal pain	14.3% (13)	18.4% (9)	9.5% (4)	0.229
Headache	2.2% (2)	0.0% (0)	4.8% (2)	0.210
Neck pain	5.5% (5)	2.0% (1)	9.5% (4)	0.177
Arm pain	1.1% (1)	2.0% (1)	0.0% (0)	1.000
Leg pain	3.3% (3)	6.1% (3)	0.0% (0)	0.246
Intensity	7.9 ± 2.1	7.5 ± 2.2	8.3 ± 1.9	0.219
Irradiation	42.9% (39)	–	–	–
Back	14.3% (13)	18.4% (9)	9.5% (4)	0.229
Arm(s)/shoulder	11.0% (10)	14.3% (7)	7.1% (3)	0.331
Neck/jaw	12.1% (11)	12.2% (6)	11.9% (5)	0.960
Chest	5.5% (5)	4.2% (2)	7.1% (3)	0.659
Abdominal	3.3% (3)	2.0% (1)	4.8% (2)	0.593
Leg	4.4% (4)	4.1% (2)	4.8% (2)	1.000
Quality				
Oppressive/tightness	34.1% (31)	34.7% (17)	33.3% (14)	0.891
Transfixing/Stabbing	16.5% (15)	16.3% (8)	16.7% (7)	1.000
Burning	4.4% (4)	2.0% (1)	7.1% (3)	0.332

The time between first symptoms and arrival in the emergency department was 22.5 ± 63.8 hours, with a range of 24 minutes minimum and 14 days maximum.

### Imaging

The imaging techniques used for AAAD diagnosis are shown in Table 4 . Computed tomography (CT) scan was the most commonly used method (93.3%). CT scan was frequently completed with a transthoracic echocardiogram (TTE) to assess heart function and plan surgery. In 3 patients who were hemodynamically unstable and were directly transferred to the operating room (OR), diagnosis was established using a transesophageal echocardiogram. Among patients who had a TTE, there were four cases of critical situations where TTE alone was performed before transferring the patient to the OR. For those, the diagnosis was confirmed by visualization of the AD after sternotomy. Chest radiography was more frequent in group 2 and could be normal or show signs of AD (e.g., enlargement of the mediastinum, tracheal deviation, enlargement of aortic contour). No magnetic resonance imaging (MRI) was performed.

**Table 4:** Imaging

Parameter	Overall ( *N* = 104)	Group 1 ( *N* = 58)	Group 2 ( *N* = 46)	*p* -Value
CT scan	93.3% (97)	94.8% (55)	91.3% (42)	0.697
TTE	53.8% (56)	50.0% (29)	58.7% (27)	0.377
TEE	2.9% (3)	3.4% (2)	2.2% (1)	1.000
Coronarography	6.7% (7)	10.3% (6)	2.2% (1)	0.130
Chest X-ray	14.4% (15)	6.9% (4)	23.9% (11)	0.014
MRI	0.0% (0)	0.0% (0)	0.0% (0)	–

Abbreviations: CT, Computed tomography; MRI, magnetic resonance imaging; TEE, transesophageal echocardiography; TTE, transthoracic echocardiography.

### Preoperative Data

Measurement of the ascending aorta revealed a mean diameter of 49.0 ± 11.6 mm and a dilation in 95.4% of patients (dilatation has been defined as > 30 mm). The maximum diameter was measured at 100 mm. Left ventricular function was overall preserved, in 90.7% of patients. “Normal” left ventricular ejection fraction (LVEF) was fixed at ≥55%. The mean LVEF was 55.6% ± 7.6. Aortic valve incompetence was found in 44.3% of the patients, including three cases of severe valvular insufficiency. Pericardial effusion was observed in 58.4% of cases and tamponade in 19.1% of cases.

Supra-aortic vessels were the most common site of extension, found in 57.7% of the cases. The dissection was limited to the ascending aorta in 22 cases (21.2%). The false lumen extended to the iliac arteries and its further branches in 49.0% of patients. Visceral arteries were involved in 45.2% of patients. There were 28.8% cases of cerebral ischemia and 25.0% cases of myocardial ischemia. Renal ischemia was also present in 9.6% of patients.

### Intraoperative Data

Once AAAD was diagnosed, all patients underwent emergency surgery. After arterial and venous cannulation, a cardiopulmonary bypass (CPB) was obtained. Concerning the cannulation sites, there was a statistically significant difference between the two groups ( Table 5 ). Femoral vessels were the preferred site of cannulation in group 1. In contrast, femoral vessels were rarely used in group 2. Instead, subclavian or axillary arteries ensured arterial flow in 56.5% of cases, and central venous canulation was used in 91.3% of cases.

**Table 5:** Cannulation site

Parameter	Overall ( *N* = 91)	Group 1 ( *N* = 49)	Group 2 ( *N* = 42)	*p* -Value
Arterial				
Femoral	52.0% (53)	73.2% (41)	26.1% (12)	<0.001
Central	12.7% (13)	8.9% (5)	17.4% (8)	0.202
Subclavian or axillary	35.3% (36)	17.9% (10)	56.5% (26)	<0.001
Venous				
Femoral	44.1% (45)	73.2% (41)	8.7% (4)	<0.001
Central	63.7% (65)	41.1% (23)	91.3% (42)	<0.001

Median sternotomy approach was used to operate all patients. Valve-sparing procedures were performed on 8 patients (five Tirone-David procedures and three Yacoub procedures). Bentall procedure was performed in 21 patients, with a higher frequency in group 2. A total of 69.0% of patients in group 1 had ascending aorta replacement alone (without arch replacement) versus 6.5% in group 2 ( *p* < 0.001). A total of 24.1% of patients in group 1 had ascending aorta replacement with hemiarch replacement versus 78.3% in group 2 ( *p* < 0.001). Total-arch replacement was performed in 3.4% of patients in group 1 versus 15.2% of patients in group 2 ( *p* < 0.05). Distal repair was rarely extended to the proximal descending aorta by the elephant trunk technique and only performed on two patients. Open distal anastomosis technique was preferred in group 2 versus under clamping technique in group 1.

Cerebral protection (CP) was assured in 51.9% of patients, the majority of whom were in group 2 (91.3%, *p* < 0.001). Antegrade cerebral perfusion (ACP) was largely preferred over retrograde cerebral perfusion (RCP), performed in 50 versus 1.9% of patients, respectively ( *p* < 0.001). RCP was obtained through mono-cannulation of the superior vena cava. Unilateral antegrade cerebral perfusion was established in 23.1% of patients, via cannulation of the subclavian artery or the brachiocephalic trunk. Bilateral antegrade cerebral perfusion (BACP) was obtained through cannulation of the brachiocephalic trunk, the subclavian artery, the left or right common carotid artery. A total of 65.2% of group 2 underwent BACP versus none in group 1. The mean duration of CP was 43.9 ± 27.8 minutes.

A period of hypothermia was established in 62.5% of patients and occurred more frequently in group 2 ( *p* < 0.001). There was no difference between the groups in mean degree of hypothermia, which was 26 ± 4°C.

Mean time of surgery was significantly different between the two groups: 5.6 hours in group 1 compared with 6.6 hours in group 2 ( *p* < 0.05). For patients that required immediate reoperation, the duration of each procedure was summed. Mean CPB and aortic cross-clamping duration were also longer in group 2 ( *p* < 0.05).

During the operation, surgeons faced 5 cases of aortic rupture and 32 cases of active bleeding, and a total of 10 patients died in the OR.

### Pathological Findings

No patient reported having a connective tissue disorder and four had a known vasculitis (three Horton's disease and one Wegener's granulomatosis). However, pathological reports of the aorta obtained after surgery suggest that 10 patients had Marfan syndrome and 4 patients had evidence of aortic wall vascular inflammation.

### Postoperative Complications

After surgery, all patients were transferred intubated to the intensive care unit for follow-up. Patients were then sent to the cardiovascular or neurology department for further surveillance until sufficient recovery. Postoperative complications are listed in Table 6 . The most frequent complications were acute renal failure and respiratory insufficiency, with no significant differences in complications between groups. Surgical revision was performed in 18 patients, mainly for bleeding, pericardial effusion, and/or tamponade.

**Table 6:** Postoperative complications

Parameter	Overall ( *N* = 91)	Group 1 ( *N* = 49)	Group 2 ( *N* = 42)	*p* -Value
Acute renal failure	31.7% (33)	29.3% (17)	34.8% (16)	0.552
Respiratory insufficiency	26.0% (27)	22.4% (13)	30.4% (14)	0.354
Cerebral ischemia/stroke	21.2% (22)	19.0% (11)	23.9% (11)	0.539
Shock	13.7% (14)	12.3% (7)	15.6% (7)	0.633
Pneumothorax/pulmonary atelectasis	7.7% (8)	6.9% (4)	8.7% (4)	0.730
Myocardial infarction	3.8% (4)	1.7% (1)	6.5% (3)	0.319
Dysphagia	7.7% (8)	5.2% (3)	10.9% (5)	0.461
Pneumonia	20.2% (21)	20.7% (12)	19.6% (9)	0.887
Revision				
Bleeding, pericardial effusion, and/ or tamponade	17.3% (18)	20.7% (12)	13.0% (6)	0.306
Sternal dehiscence	4.8% (5)	3.4% (2)	6.5% (3)	0.653
Mediastinitis	1.9% (2)	0.0% (0)	4.3% (2)	0.193

### Late Reoperations

**Fig. 1:**
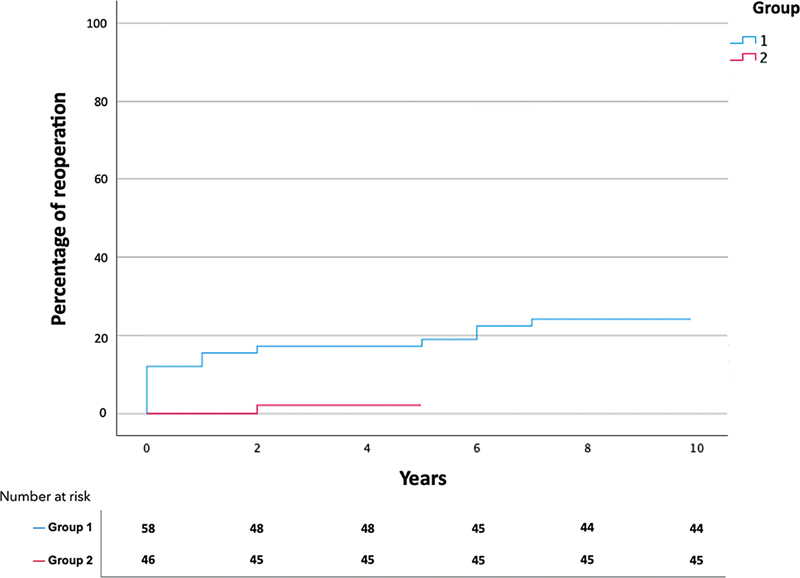
Percentage of reoperations in both groups. Blue curve is group 1. Red curve is group 2. There was significantly less reoperation in group 2, but the follow-up period was shorter in this group.

We observed a marked difference in the percentage of reoperations between the two groups. Only 1 of the 15 patients who required reoperation belonged to group 2 ( Fig. 1 ). The follow-up period was also significantly longer in group 1, with 6.6 ± 4.4 versus 2.4 ± 2.1 years in group 2 ( *p* < 0.001).

Causes of late reoperation, thus after discharge from the hospital, are shown in Table 7 . Aortic dilation was significantly lower in group 2 at 2.2%, compared with 19.0% in group 1. Recurrent AD was caused by a patent intimal tear or the occurrence of a new intimal tear.

**Table 7:** Cause of reoperation

Parameter	Overall ( *N* = 104)	Group 1 ( *N* = 58)	Group 2 ( *N* = 46)	*p* -Value
Total number of reoperations	14.4% (15)	24.1% (14)	2.2% (1)	0.002
Aortic dilatation (aneurysm, pseudoaneurysm)	11.5% (12)	19.0% (11)	2.2% (1)	0.008
Recurrent aortic dissection	7.7% (8)	12.1% (7)	2.2% (1)	0.074
Aortic valve insufficiency	3.8% (4)	6.9% (4)	0.0% (0)	0.128

### Short-Term and Long-Term Mortality after Surgery

Mortality rates are shown in Table 8 . Aorta-related mortality is defined as death from any AD complication, including aortic rupture, malperfusion syndrome, tamponade, redissection, or extension of the dissection, during first hospitalization, or rehospitalization for AD (death during surgery or death from postoperative complications). Causes of short-term death were all considered aorta-related events. Other cardiovascular-related events include nonaorta-related events such as acute coronary syndrome, stroke, arrhythmia, heart failure, and rehospitalization for nonaorta-related causes. Noncardiovascular death concerned all other causes of death.

**Table 8:** Mortality

Parameter	Overall ( *N* = 104)	Group 1 ( *N* = 58)	Group 2 ( *N* = 46)	*p* -Value
Overall mortality	38.5% (40)	43.1% (25)	32.6% (15)	0.275
30-d mortality	21.2% (22)	22.4% (13)	19.6% (9)	0.724
Long-term mortality	17.3% (18)	20.7% (12)	13.0% (6)	0.306
Aorta-related event	4.8% (5)	3.4% (2)	6.5% (3)	0.653
Other cardiovascular-related event	3.8% (4)	6.9% (4)	0.0% (0)	0.128
Nonvascular related event	8.7% (9)	10.3% (6)	6.5% (3)	0.728
Follow-up, mean ± SD (y)	4.8 ± 4.1	6.6 ± 4.4	2.4 ± 2.1	<0.001

Abbreviation: SD, standard deviation.

**Fig. 2:**
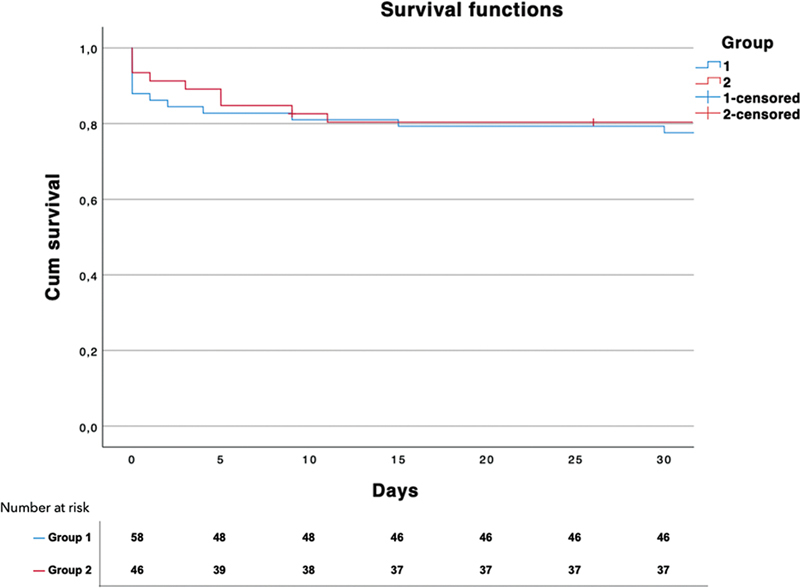
Kaplan–Meier estimates of survival comparing 30-day mortality of group 1 (blue curve) and group 2 (red curve).

**Fig. 3:**
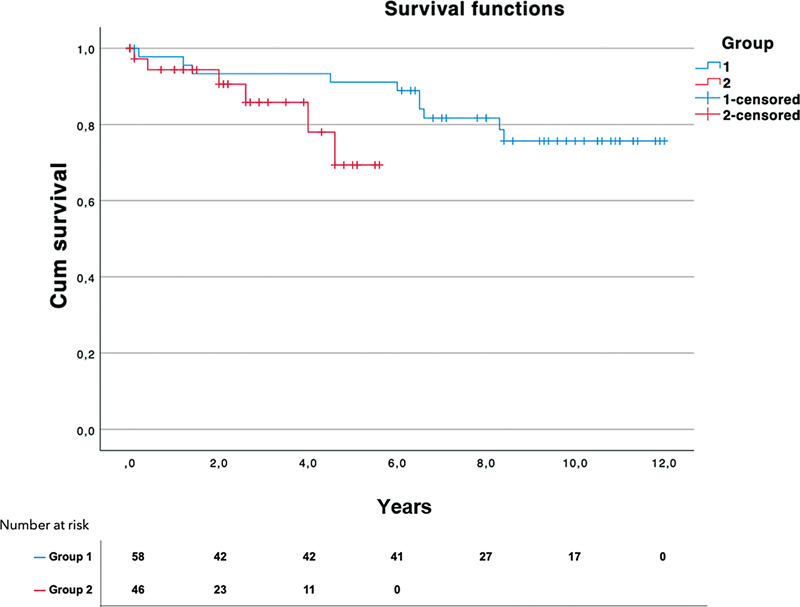
Kaplan–Meier estimates of survival comparing long-term mortality of group 1 (blue curve) and group 2 (red curve).

There was no significant difference between the two groups in terms of mortality. The survival curves are illustrated in Figs. 2 and 3 .

The follow-up period was significantly longer in group 1, with 6.6 ± 4.4 years compared with 2.4 ± 2.1 in group 2 ( *p* < 0.001).

## Discussion

In this study, demographics and patients' profiles were overall similar between both groups except for smoking and diabetes. This may be explained by missing data on patients' records or unfound information. The mean age was comparable to that reported in the International Registry of Acute Aortic Dissection (IRAD). 4 Women were significantly older than men as seen in the German Registry for Acute Aortic Dissection Type A. 5 Hypertension was the most frequent risk factor, found in 55.8% of patients. The IRAD reported 69.3% of hypertension, which is higher than our rate. 4 Patients' history revealed 4.8% of known aortic aneurysms, 8.7% of prior aortic or cardiac surgery versus, respectively, 12.4% and 15.9% in the IRAD. Marfan syndrome was more frequent in the studied population than in the IRAD according to pathological findings, but genetic confirmations were unavailable in our study.

Acute AD typically presents with abrupt and sharp chest pain, often described as the “worst ever” by patients. 4 6 Indeed, sudden onset of symptoms and pain were the most frequent features in our study. However, those characteristics overlap with other more common conditions, which can lead to misdiagnosis and management delay. 2 6 In our study, some patients were initially diagnosed with myocardial infarction, pulmonary embolism, stroke, or even acute pancreatitis after clinical examination. An AD was frequently discovered on further investigation for those other conditions. Considering the high mortality rate of AD, time is an important variable to consider, if not the most important. According to the IRAD, features associated with delay in the diagnosis were female sex and atypical symptoms. 7

Diagnosis of AD is confirmed by imaging techniques. Transesophageal Echocardiography (TEE), CT, and MRI have comparable specificity and sensitivity, but CT is the most used technique as it offers great images in a short delay of time and is rapidly available. MRI is the most performant, but its long acquisition time, poor availability, and contraindications limit its use. Echocardiography has the advantages to provide rapid assessment and being widely accessible. TEE has the lowest acquisition time, but its accuracy is operator-dependent. It is usually performed on unstable patients and is a great alternative for patients with contradictions for CT. TTE is useful in emergency situations to assess high-risk signs such as pericardial effusion or tamponade; however, its sensitivity for AD is lower than other techniques. 1 8 In our study, nearly every patient had a CT (92.3%). No patient had an MRI, and a majority of patients (67.3%) had an echocardiography. Unlike group 1, ultrasound and chest X-rays were more frequently used for AD assessment in group 2 ( *p* < 0.05). Chest X-rays are usually performed routinely to investigate chest pain in our institution, since it is rapidly available and can help to rule out alternative diagnosis, but this modality has a poor specificity for AD. Imaging also provides important preoperative data to plan the surgery for each patient. In addition to information concerning repercussions of AD on heart pump function (aortic valve function, LVEF, pericardial effusion, tamponade), organ malperfusion, and extent of AD, imaging also helps to assess possible cannulation sites for CPB to anticipate any vascular access problem such as the presence of atherosclerotic plaques. 9

CPB can be performed through different approaches; historically, femoral arterial cannulation was the standard procedure. 10 11 Over time, axillary cannulation has become the preferred arterial cannulation site [Malaisrie]. Notably, because several studies have shown that axillary cannulation reduces short-term mortality, induces less neurological deficit, and facilitates cerebral perfusion. 9 12 13 Moreover, axillary arteries are less prone to atherosclerotic plaques. 10 Since the introduction of a standardized protocol, our study has shown that subclavian/axillary arterial cannulation frequency was significantly higher than before. Among patients of group 2 whose axillary artery was inaccessible, the alternatives were femoral or central cannulation. Concerning venous cannulation, our study has shown that central venous cannulation was preferred in group 2 (93.3%). In group 1, venous outflow was preferably accomplished through the femoral vein (74.5%).

To prevent neurological lesions during aortic surgery, several methods have been developed since the first aortic arch replacement by De Bakey and Cooley in 1955. 14 In our study, CP was ensured by selective cerebral perfusion or HCA or a combination of both. The results have shown that hypothermia and CP were more frequent in patients of group 2. HCA was practically always associated with ACP. Cooling down the patient allows the reduction of the brain's activity and thus its metabolic needs. However, this technique is limited in time since the brain's metabolism never reaches zero even at the lowest temperature. 14 15 16 In this regard, there is a time limit on the use of HCA. When surgery is expected to exceed this time, the adjunction of CP provides supplementary protection to the brain. 16 ACP has become the technique of choice for brain protection during aortic surgeries as seen in our study.

Our study showed a higher tendency for Bentall procedure in group 2. The literature is still debating on the outcomes of aortic root-sparing repair versus root replacement but several studies have shown no difference in terms of short- or long-term outcomes between those two procedures. 1 17 18 19 Aortic root replacement is indicated when there is a significative dilatation impairing the function of the aortic valve, when the dissection involves the aortic root, when the initial tear is located within the root, or when the patient has a connective tissue disorder. 10 11 20 It reduces the risk of reoperation for late aortic root dilatation and aortic regurgitation as highlighted by our study, with fewer cases of aortic dilatation in group 2 despite the follow-up period being shorter in this group compared with group 1 ( *p* < 0.001). 1 Concerning distal repair, our study showed more extensive reparation in group 2 with more hemiarch and total-arch replacement than in group 1. Ascending aortic replacement alone is technically easier and quicker to perform. 1 21 However, it was associated with a greater risk of distal dilatation and thus of late reoperations. 1 21 A meta-analysis comparing hemiarch and total arch replacement conducted by Poon et al 22 showed similar short-term and long-term mortality rates between both techniques, as well as reoperation rates. Hemiarch replacement was associated with shorter surgery, aortic cross-clamp, and circulatory arrest durations, and thus favorable early morbidity.

Distal anastomosis was either accomplished by closed or open techniques. Each technique has its own advantages and risks. Our study shows a preference for open distal anastomosis in group 2 as suggested by current literature. Open distal repair has been routinely used as it allows greater visualization of the aorta, a more extensive repair of the aorta, and a decreased risk of injury caused by the clamp reduces the risk of a patent false lumen. However, this technique requires HCA often associated with CP, lengthening the duration of surgery. A closed technique is much quicker because it can be performed without cooling and the establishment of CP. Although several studies have shown comparable mortality between both techniques, open distal anastomosis remains the currently preferred technique. 21 23 24 25

According to the IRAD, 4 the 30-day mortality rate of patients treated surgically for AAAD was around 26%, slightly higher than our results which were 21.2%. Our study showed an increase in surgery duration in group 2 since more extensive techniques were adopted, requiring the establishment of CPB, CP, and hypothermia. Revision surgery was required for 15 patients in total, among them only 1 was from group 2. Since the follow-up stopped at the end of the study, i.e., on December 31, 2021, group 2 had a shorter follow-up, but we can already see a marked difference between both groups after 2 years of follow-up in Fig. 1 .

### Limitations

This study includes the usual limitations of retrospective studies. All data were extracted from electronic patient records only. The follow-up period of both groups is unequal. This study is focused on a single health center, providing a small study population.

## Conclusion

Our institution's current surgical protocol is consistent with the latest strategies suggested by several studies. Our results did not show a significant difference in mortality since the introduction of the standardized protocol, but there were significantly fewer reoperations and aortic dilatation rates. Converting AAAD operations from a “surgeon-tailored” to a “patient-tailored” approach is feasible and may help to get better results in small-volume centers like ours.
